# Person-organization fit and job burnout of researchers during the COVID-19 pandemic: Heterogeneity in eleven countries

**DOI:** 10.1371/journal.pone.0302296

**Published:** 2024-05-09

**Authors:** Xiao Liu, Cathy Ping Xie

**Affiliations:** 1 Institute of Education, Nanjing University, Nanjing, Jiangsu, P. R. China; 2 Maestro International School, Harbin, P. R. China; St John’s University, UNITED STATES

## Abstract

To manage the negative impact of job burnout for the researchers, especially during COVID-19 pandemic, is not easy. Thus, it is essential for educational institutions to provide them with the support they need to improve the person-organization (P-O) fit. Drawing upon the data from the Nature’s Global Survey initiated in 2021, this paper analyzed 2,424 effective samples from eleven countries in the world to investigate how P-O fit impacts researchers’ job burnout in different countries and their career stages during the COVID-19 pandemic. The findings show that both organizational support and P-O fit have significantly assisted researchers in all career stages to reduce job burnout. Moreover, P-O fit has a greater inhibitory effect on job burnout than organizational support. However, when resources are relatively scarce in some developing countries, it is more important to provide organizational support for researchers. Therefore, in order to improve the efficiency of organizational support and reduce researchers’ job burnout, those aspects which are less fit but helpful should be increased appropriately. Moreover, it implies that it would be significant to emphasize the differentiated and career-stage-sensitive resources and support to researchers in different countries in the post-pandemic era to improve researchers’ well-being and organizational performance.

## Introduction

Job burnout, originated from “staff burnout” when Freudenberg first produced in 1974 as a unique stress response syndrome, is a negative attitude and feeling of individuals who work with extreme mental stress, tiredness and indifference to work for a long time [1]. Studies have confirmed that burnout can lead to various detrimental outcomes, such as anxiety and depression [2], which would damage employees’ physical and mental health [3], as well as reduce job involvement and engagement of employees [4], which resulted in the lower level of job satisfaction [5], an increase in employee turnover [6], and in turn affects organizational performance [7]. Therefore, job burnout has always been an important issue of great concern to researchers and practitioners around the world.

Existing research on burnout has focused on identifying factors that may prevent or reduce this behavior with appropriate interventions. A large number of studies on job burnout have found that factors, including individual, job, occupational and organizational aspects, interconnectedly affect the occurrence of job burnout [2]. Among them, organizational support, proposed by Eisenberger and his colleagues in 1986, which refers to employees’ comprehensive perception of how the organization value their own contributions and well-being, is the most common [8]. Moreover, Chen et al. examined the correlation between job burnout and job satisfaction through perceived organizational support and work engagement, and proposed that perceived organizational support mediated the association between job burnout and work engagement and the connection between job burnout and job satisfaction [9]. Afterwards, Maslach and her colleagues believed that job burnout was not caused by work or individuals unilaterally, but by the person-organization (P-O) fit. P-O fit, originated from attraction-selection-attrition (ASA) framework, is broadly conceptualized as the compatibility between people and organization [2, 10].

Gradually, P-O fit and organizational support have gradually improved and begun to expand from the field of organizational theory to the field of work. The impact of organizational support and P-O fit on work has permeated many aspects of work, including employee job search, job engagement, pressure, job satisfaction, and job performance. At present, when scholars discuss the factors that affect employees’ work, organizational support and P-O fit have become two dominant factors that cannot be ignored in this field.

The problem of job burnout among researchers is increasingly common worldwide [11]. In addition, the outbreak of the COVID pandemic has caused unprecedented challenges to researchers globally. Studies found that COVID-19 has disrupted research worldwide, leading to lost research time and increased anxiety amongst researchers [12–14]. Given the uncertainties and challenges that the COVID-19 pandemic has caused to researcher burnout, it is more important for organizations to support researchers, especially to meet their work needs, so that research could be carried out smoothly and scientific output would be promoted. However, the importance and motivation of occupational demands would change as the career stage develops [5].

Therefore, it is worth addressing the investigations as follows:

1. Since COVID-19, what would be the percentage of job burnout among researchers in various countries?
2. What would be the organizational support, individual needs, and person-organization (P-O) fit that researchers receive?
3. Is it likely that organizational support and person-organization (P-O) fit reduce the job burnout of researchers?
4. Which aspects of organizational support have less contribution to the organization but greater impact upon job burnout?

This paper utilizes the data of an online survey on the job burnout of global researchers organized by Nature in 2021 to investigate and address the questions above. **Theoretically**, this study is likely to strengthen the importance of the P-O fit theory and primarily provides the evidence-informed human resource management of the application of the P-O fit theory in the context of the pandemic, which was measured by the compatibility between personal demands of researchers and organizational support perceived by researchers in eight dimensions in eleven countries. It contributes to our understanding of the fitness between researchers and organizations. Moreover, **pragmatically**, the findings of this study also have a number of important implications for future practices. This paper would provide a reference for how to reduce researchers’ job burnout at the organizational level in the post-pandemic era. Also, this study would reinforce the existing literature to emphasize the differentiated and career-stage-sensitive resources and support to researchers in different countries.

## Materials and methods

### Theoretical Framework

**Fig 1:**
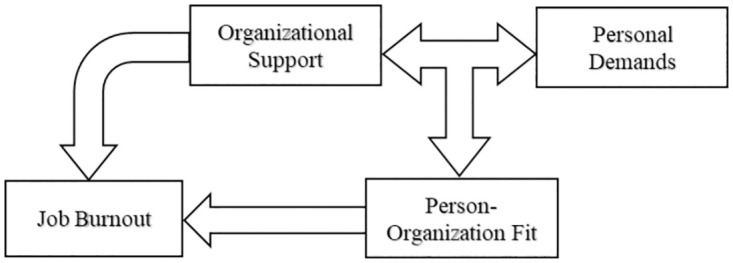
Theoretical framework of the impact of P-O fit on job burnout.

The theoretical framework of this study is constructed as Fig 1 shows. Specifically, based on the P-O fit conceptual framework of Kristof [11], this study would explore the P-O fit of researchers in eleven countries by the compatibility between personal demands and organizational support to investigate how the organizational support would impact researchers’ job burnout.

### Data

The data in this paper are from the Nature’s Global Career Development Survey initiated by The Nature in 2021, supported and collected by Shift Learning, a market-research company based in London. This survey, which education research specialists Shift Learning are conducting on behalf of Springer Nature, aims to explore the job burnout of researchers during the COVID-19 pandemic as well as their expectations on their career development in the long term. Authors have no access to information that could identify individual participants during or after data collection.

The survey, in five languages of English, Mandarin, Spanish, French and Portuguese, which was circulated from 7 June to 11 July, 2021 by e-mails, drew responses from more than 3,210 scientific researchers around the world. The underlying dataset is available from https://figshare.com/s/834fa8d8baf36f2e5c97. To address the issue of sample diversity and numbers, several filters were implemented: 1) the sample number is at least over 70; 2) the respondents are excluded if they are not related with research itself; 3) the respondents are the full-time researchers. Therefore, we draw upon the data of the full-time researchers from eleven countries (United States, United Kingdom, Germany, China, Canada, Spain, Brazil, Australia, India, Italy & France) with 2,424 samples in total as Table 1 shows. Among them, samples are largely aged 31 to 40 (38.83%), among which females account for 50.99% and more than 42% are in their early career stages. Also, 23.14% of participants are post-docs. A large proportion of respondents are in biomedical and clinical sciences (40.35%). 79.55% obtained the Higher Degree.

**Table 1:** Sample description.

Country	Sample size	Position / Role	Percent
United States	1,002	Postdoctoral fellow / research associate	23.14%
United Kingdom	433	Research / staff scientist	18.81%
Germany	184	Full professor	9.48%
China	146	Assistant professor	8.83%
Canada	108	Associate professor	8.46%
Spain	108	Technician / technical manager	8.00%
Brazil	105	Middle or senior management	3.63%
Australia	101	Data analyst/scientist	2.85%
India	88	Lecturer / instructor or other primarily teaching job	2.68%
Italy	78	Project manager	2.43%
France	71	Consultant	2.22%
Age	Percent	Research Director / VP of research	1.73%
25-	4.79%	Clinician	1.57%
26–30	12.70%	Professional	1.38%
31–40	38.83%	Science communications	1.20%
41–50	20.96%	Business development	0.74%
51–60	13.90%	Other	2.85%
61+	8.82%	Field of work	Percent
Gender	Percent	Biomedical and clinical sciences	40.35%
Female	50.99%	Ecology and evolution	7.17%
Male	46.29%	Health care	6.77%
other	2.72%	Chemistry	6.67%
Career Stage	Percent	Social sciences	6.44%
Early career	42.24%	Geology and environmental science	6.19%
Mid-career	38.94%	Engineering	5.20%
Late career	18.81%	Agriculture and food	4.79%
Degree	Percent	Physics	4.74%
Higher Degree (PhD/MD/JD etc)	79.55%	Computer science and mathematics	3.63%
Masters’ level (MSc/MA/MBA)	12.04%	Astronomy and planetary science	1.94%
Undergraduate/Bachelor’s degree or equivalent	8.41%	Other science-related field	6.11%

### Variables

#### Independent variables: Person-organization fit and organizational support

**Fig 2:**
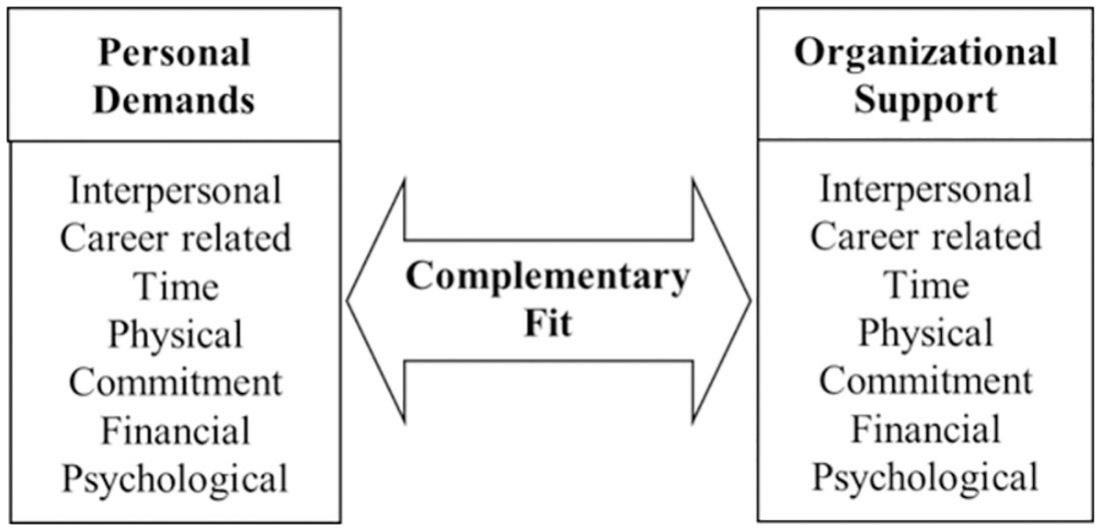
The framework of the measurement of P-O fit.

In the measurement of P-O fit, initially, Muchinsky et al. proposed two dimensions: supplementary fit and comprehensive fit to measure [15]. Although these two distinctions have been discussed frequently by authors, they have rarely been integrated. For example, most empirical investigations have defined fit from only one perspective, while ignoring the existence of the others. These multiple conceptualizations of fit reasonably explain the variety of operationalizations that have been used to examine P-O fit, yet to integrate the variety of P-O fit conceptualizations, a comprehensive definition is needed. Afterwards, Kristof [11] integrated the two distinctions above and posited that organizations supply, such as financial, physical, and psychological resources, as well as the task-related, interpersonal, and growth opportunities that are demanded by employees. When these organizational resources meet employees’ demands, needs-supplies fit is achieved. Similarly, organizations demand contributions from their employees in terms of time, effort, commitment, knowledge, skills, and abilities. Demands-abilities fit is achieved when these employee resources meet organizational demands. Drawn upon the studies by Muchinsky and Kristof, the P-O fit in this study is measured at the individual level, and defined as the compatibility between personal demands and organizational support [10, 15]. Among them, individual needs are the degree of those of scientific researchers for various organizational support. Organizational support refers to the degree to which researchers feel that the organization values their contributions and cares about their well-being [16, 17]. As Fig 2 shows, Nature’s Survey investigated the organizational support perceived by each researcher and the importance of their personal demands in seven dimensions: interpersonal, career related, time, physical, commitment, financial, and psychological. To summarize, due to the existing data set available from Nature, we would only focus on the complementary fit and do not discuss the supplementary fit in this paper.

As the 28 items on organizational support and the same number of personal needs are a one-to-one correspondence in the survey, the P-O fit of each item is then calculated by the following formula:

$$POF_{i,n}=\frac{OS_{i,n}}{PD_{i,n}}\times 100\%$$ (1)

In formula (1), POF*i*,*n* represents the i-th person-organization fit of the n-th researcher. OS*i*,*n* represents the satisfaction of the i-th organizational support of the n-th researcher. PD*i*,*n* represents the importance of the i-th personal demand of the n-th researcher.

The specific 28 items on organizational support and personal demands are shown in Table 2. All survey questions utilized a 5-point Likert scale. Among them, the organizational support is measured by satisfaction from extremely dissatisfied, somewhat dissatisfied, neither satisfied nor dissatisfied, somewhat satisfied to extremely satisfied, and they are assigned a value of 1 to 5 in turn, while the personal demands are measured by the degree of feeling: somewhat unimportant, not at all important, neither important nor unimportant, somewhat important, extremely important, which are assigned a value of 1 to 5 accordingly. Moreover, the values of Cronbach’s Alpha in each dimension are all above 0.8, suggesting it is a reliable measure of organizational resources and personal demands during the COVID-19 pandemic.

**Table 2:** 28 items to measure the organizational support / personal demands.

Dimension	Items	Abbreviation
Interpersonal	Communication with supervisor	CWS
	Relationship with colleagues (including social events organized either by the company or amongst colleagues)	RWC
Career related	Career advancement opportunities	CAO
	Access to workplace-sponsored training and seminars	ATW
	Job security	JS
	Opportunity to work on interesting projects	OTW
	Amount of guidance from supervisor	AOG
	Ability to choose remote/hybrid working options	ATC
	Work independence	WIN
	Recognition for achievements	RFA
Time	Amount of time for research	AOT
	Work/life balance	WLB
	Compatibility of job with family life/raising children	COJ
	Time off (e.g. vacation, bank holidays, personal days, sick days)	TO
	Commuting time	CT
	Total hours worked	THW
Physical	Workplace facilities and comfort	WFA
	The safety of the work environment / workplace	TSO
Commitment	Diverse representation within management and leadership (e.g. race, gender, sexual orientation)	DRW
	Organization’s commitment to a diverse and inclusive workplace	OCD
	Organization’s commitment to environmental sustainability	OCE
Financial	Salary/compensation	SC
	Availability of funding	AOF
	Financial resources of organization	FRO
	Benefits (e.g. health and dental insurance, retirement plan)	BEN
Psychological	Personal sense of accomplishment	PSO
	Work interest	WI
	Meaningfulness of job	MOJ

#### Dependent variable: Job burnout

In the measurement of job burnout, Maslach’s designed occupational burnout scale has been widely recognized. She has developed the Maslach Burnout Inventory (MBI) including MBI-Human Service Survey (MBI-SS), MBI-Educators Survey (MBI-ES) and MBI-General Survey (MBI-GS). Drawn upon the MBI-GS developed by Maslach et al. [2], Nature’s Survey has revised this scale given the context of researchers, with three items deleted and thirteen retaining ones, which allowed researchers to assess the frequency of different dimensions of burnout [18]. Consistent with other research, thirteen items are also induced to three dimensions after factor analysis: emotional exhaustion, depersonalization, and reduced personal accomplishment, with thirteen items (see Table 3), using a five-point Likert scale, ranging from never, rarely, occasionally, frequently and always. What’s more, factor analysis calculated the factor scores of each individual in three dimensions. The higher the score, the stronger the job burnout in this dimension. The confirmatory factor analysis shows that χ2/df = 6.098, root mean square error of approximation (RMSEA) = 0.08, standard root mean residual (SRMR) = 0.06, and the reliability coefficient Cronbach’s α was 0.90, and all the fitting indexes reached an acceptable level. RMSEA and SRMR are both indicators of model fitness. When RMSEA<0.10 and SRMR<0.10, it indicates that the model has a good fitting effect.

**Table 3:** Job burnout scale.

Dimension	Items
Emotional Exhaustion	I feel run down and drained of physical or emotional energy
	I feel unmotivated and lacking will to complete parts of my job
	I feel under an unpleasant level of pressure to succeed
	I feel that there is more work to do than I practically have the ability to do
	I feel as if I don’t have enough time to plan experiments or projects
Depersonalization	I am harder and less sympathetic with people than perhaps they deserve
	I am easily irritated by my colleagues and team
	I feel that organizational politics or bureaucracy frustrate my ability to do a good job
	I feel isolated and that I have no one to talk to
Reduced personal accomplishment	I feel unappreciated by my colleagues
	I feel that I am achieving less than I should
	I feel that I am not getting what I want out of my job
	I feel that I am in the wrong organization or the wrong profession

#### The moderator: Career stage

This study, initiated by Nature employed a three level career stages self-judged by researchers, namely early-career, mid-career and late career to broadly explore how the researchers’ demands have been achieved in relation to organizational resources.

### Analysis

**Fig 3:**
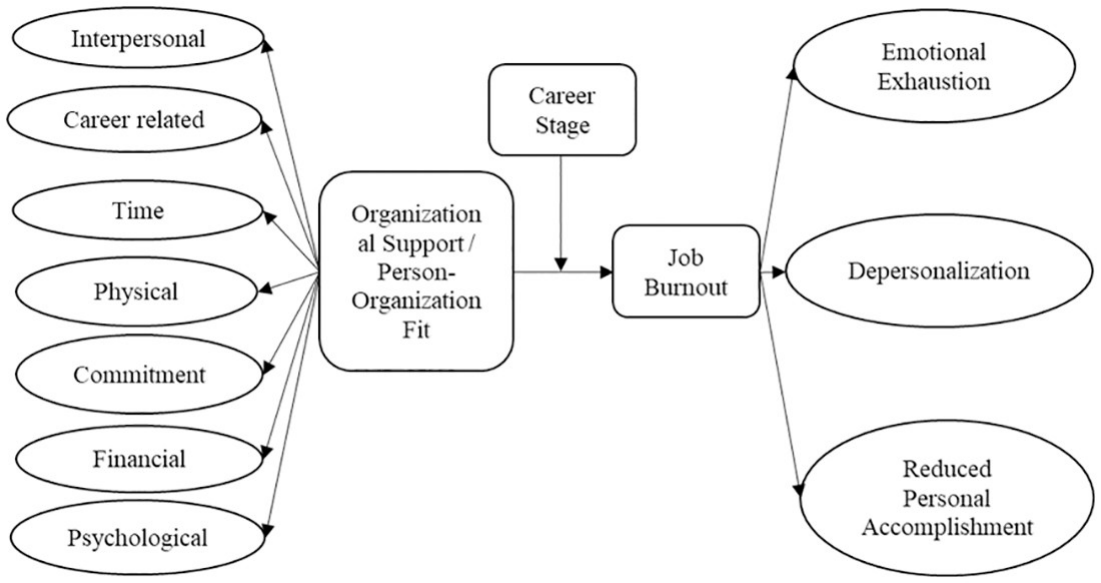
Statistical model of the impact of P-O fit on job burnout.

In order to analyze the impact of organizational support and P-O fit on job burnout and compare the magnitude of the impact, Amos is adopted for path analysis. Based on the Theoretical Model shown in Fig 1 and the variable settings, the econometric model is shown in Fig 3.

## Results

### The Job Burnout and P-O Fit of Researchers during COVID-19

**Fig 4:**
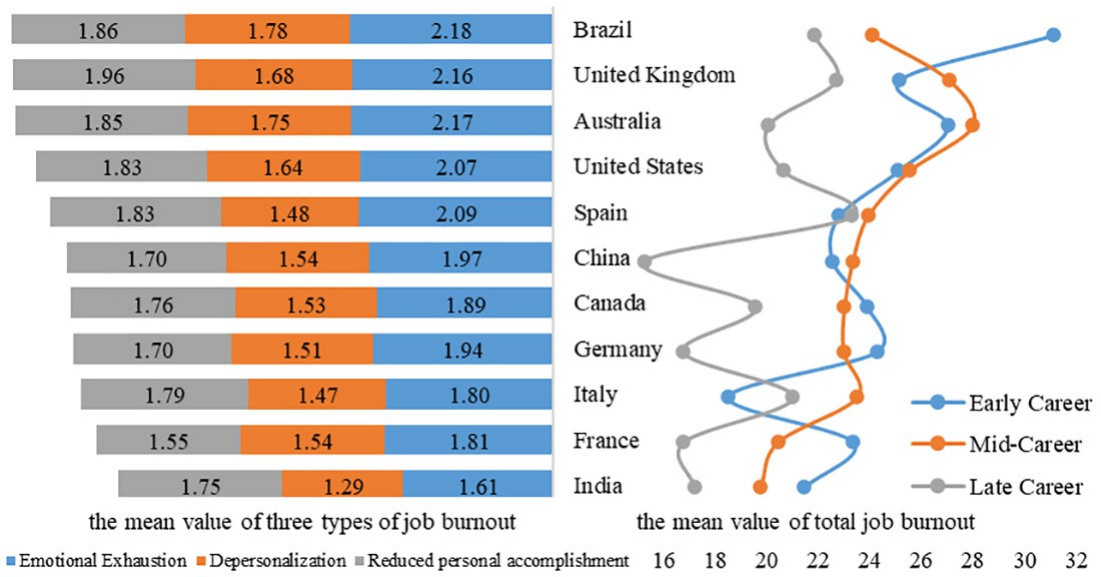
Job burnout of researchers in different career stages in eleven countries.

As the left half of Fig 4 shows, the mean value of three dimensions of job burnout were calculated separately to describe in what degrees of job burnout of researchers in eleven countries during COVID-19. Relatively speaking, among the three dimensions of job burnout, the emotional exhaustion of researchers is the most serious, while the depersonalization is the least. In 11 countries, the job burnout of researchers in Brazil, Britain and Australia is more serious while that in India, France and Italy is relatively the least.

In regard to the difference of career stages on the right half of Fig 4, the situation varies in different countries. In Brazil, India, Canada, Germany, and France, the job burnout of researchers in late career is the lowest whilst researchers in early career have the highest job burnout. However, among four countries of United Kingdom, Australia, United States, and China, the job burnout of researchers in late career is the lowest while researchers in mid-career have the highest job burnout. Meanwhile, in Spain and Italy, the job burnout of researchers in mid-career is the lowest while researchers in mid-career have the highest job burnout.

What’s more, the P-O fit of researchers in eleven countries is also calculated as Table 4 shows. The overall P-O fit of researchers in eleven countries is 77.76%. In the three career stages, the P-O fit of researchers in late career is the highest (80.76%), while the mid-career is the lowest (76.13%). Among the 28 items, the fitness of commuting time (CT), work independence (WIN) and ability to choose remote/hybrid working options (ATC) are the highest while the fitness of career advancement opportunities (CAO), availability of funding (AOF) and diverse representation within management and leadership (DRW) are the lowest. What’s more, out of 28 items, 8 items, i.e. career advancement opportunities (CAO), work independence (WIN), recognition for achievements (RFA), availability of funding (AOF), Financial resources of organization (FRO), communication with supervisor (CWS), amount of time for research (AOT) and commuting time (CT), had the highest P-O fit in the early career stage rather than the late career stage.

**Table 4:** The P-O fit of researchers in different career stages in eleven countries.

Dimension	Item	Total	Early Career	Mid-career	Late Career
Career Related	Amount of guidance from supervisor	79.50%	81.37%	75.34%	84.02%
	Choose remote/hybrid working options	86.91%	87.15%	85.71%	88.86%
	Access to training and seminars	78.12%	78.83%	76.50%	80.06%
	Career advancement opportunities	61.67%	63.73%	58.73%	63.28%
	Work independence	87.48%	88.46%	86.27%	87.82%
	Job security	73.98%	68.33%	74.25%	87.38%
	Opportunity to interesting projects	81.60%	82.00%	80.42%	83.14%
	Recognition for achievements	72.04%	73.73%	69.89%	72.73%
Commitment	Diversity within management leadership	67.36%	66.49%	65.79%	73.49%
	Diverse and inclusive workplace	74.80%	74.31%	74.15%	77.57%
	Environmental sustainability	74.34%	72.89%	74.37%	77.72%
Financial	Availability of funding	67.22%	69.54%	65.50%	64.92%
	Benefits	79.06%	77.29%	77.78%	86.16%
	Financial resources of organization	74.65%	78.59%	71.63%	71.86%
	Salary/compensation	73.30%	70.33%	72.58%	82.01%
Interpersonal	Communication with supervisor	78.33%	80.28%	75.64%	79.05%
	Relationship with colleagues	82.07%	81.09%	82.28%	84.01%
Physical	Safety of the work environment	86.12%	87.01%	84.07%	88.60%
	Workplace facilities and comfort	82.64%	83.62%	81.12%	83.67%
Psychological	Meaningfulness of job	82.70%	82.57%	81.38%	85.74%
	Personal sense of accomplishment	77.44%	76.45%	76.17%	82.26%
	Work interest	83.12%	82.90%	81.86%	86.24%
Time	Amount of time for research	75.63%	81.47%	70.32%	72.76%
	Compatibility of job with family	72.67%	69.51%	72.15%	81.42%
	Commuting time	88.97%	90.26%	87.98%	88.02%
	Total hours worked	78.45%	80.20%	75.35%	81.23%
	Time off	83.24%	81.90%	82.62%	87.99%
	Work/life balance	73.76%	73.19%	71.88%	79.25%
Total		77.76%	77.98%	76.13%	80.76%

**Fig 5:**
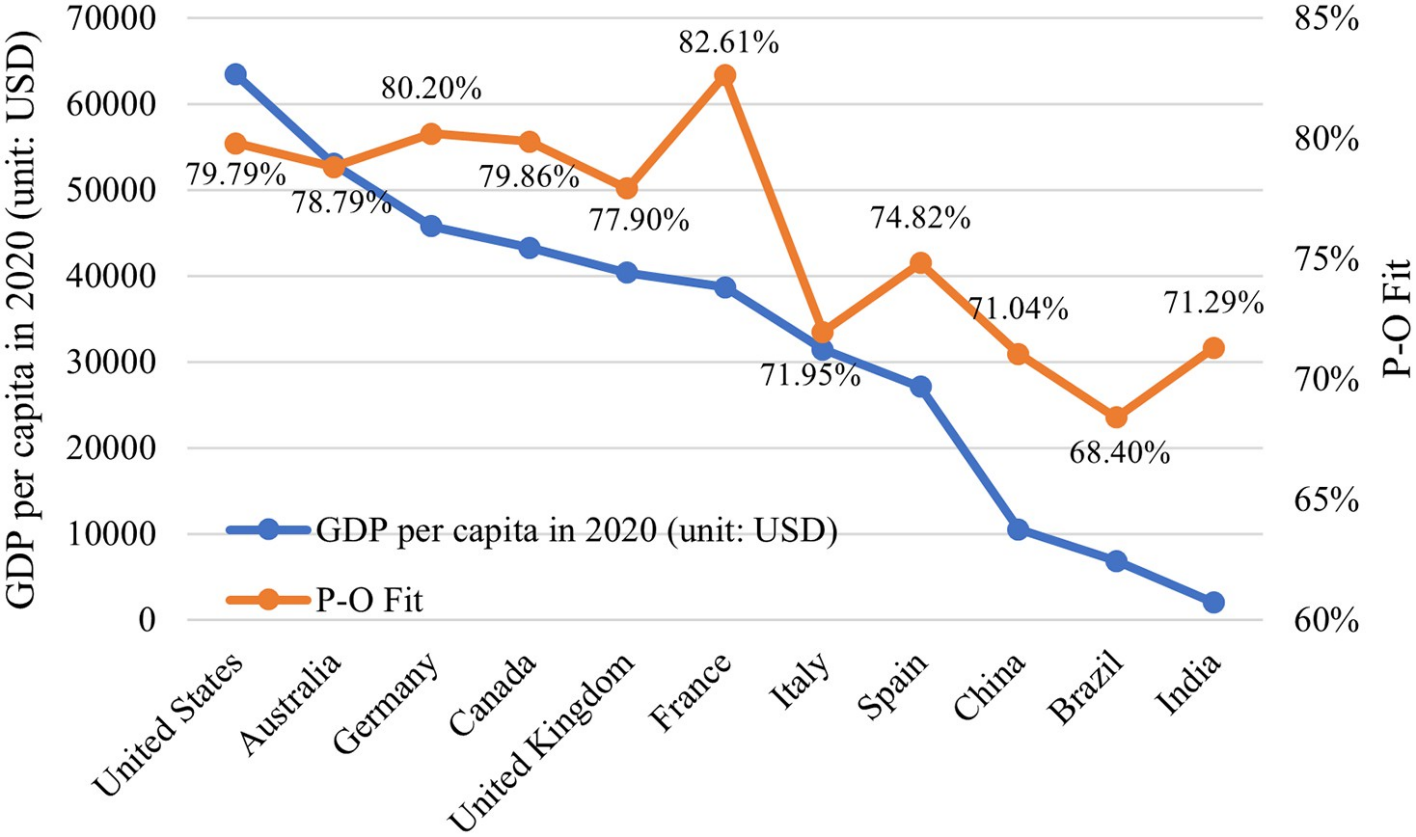
The correlation between P-O fit and GDP in eleven countries.

From the P-O fit of researchers in each country, as shown in Fig 5, among the 11 countries, the P-O fit of researchers in France is the highest (82.16%), while the P-O fit in Brazil is the lowest (68.40%). Moreover, the difference of P-O fit between countries is related to the economic development level of each country. As Fig 5 shows, the P-O fit in these eleven countries is related to their GDP per capita in 2020. Countries with high GDP per capita have a high degree of P-O fit, while countries with low GDP per capita, the P-O fit of researchers is also relatively low.

### Organizational support, P-O fit and job burnout of researchers

Based on the theoretical framework shown in Fig 1, the path model, as shown in Fig 3, was constructed to present the relationship between organizational support and researchers’ job burnout. The result shows a good model fit in Table 5. Regarding the standardized estimates and the significance of standardized estimates, organizational support has significantly reduced the job burnout of researchers in all career stages. What’s more, organizational support facilitates the mid-career researchers the most (-0.667), early career researchers second (-0.666) and late career ones the least (0.630). This is likely to be explained by the low job burnout of researchers in late career. From the perspective of three specific aspects of job burnout, organizational support is the most helpful for depersonalization.

**Table 5:** Path analysis result of the impact of P-O fit on job burnout.

	Organizational Support→Job Burnout				P-O fit→Job Burnout			
	Total	Early Career	Mid-Career	Late Career	Total	Early Career	Mid-Career	Late Career
Job Burnout	-0.661***	-0.666***	-0.667***	-0.630***	-0.741***	-0.761***	-0.733***	-0.733***
Emotional Exhaustion	-0.518***	-0.526***	-0.510***	-0.507***	-0.577***	-0.580***	-0.575***	-0.593***
Depersonalization	-0.678***	-0.683***	-0.663***	-0.697***	-0.604***	-0.632***	-0.589***	-0.605***
Reduced personal accomplishment	-0.532***	-0.474***	-0.586***	-0.532***	-0.796***	-0.798***	-0.789***	-0.810***
RMSEA	0.020	0.026	0.022	0.027	0.020	0.025	0.020	0.030
Default model	1799.983	1540.096	1418.06	1344.901	1710.079	1495.618	1338.431	1369.917
Saturated model	1804.00	1722.00	1722.00	1722.00	1722.00	1722.00	1722.00	1722.00
Independence model	52306.08	21463.28	21504.95	10743.74	48859.45	19929.96	20404.22	9894.00
Normed fit index (NFI)	0.979	0.956	0.962	0.931	0.976	0.951	0.96	0.914
Relative fit index (RFI)	0.969	0.936	0.944	0.898	0.967	0.933	0.945	0.882
Incremental fit index (IFI)	0.989	0.982	0.988	0.982	0.988	0.981	0.989	0.973
Tucker-Lewis index (TLI)	0.984	0.973	0.982	0.973	0.983	0.973	0.985	0.963
Comparative fit index (CFI)	0.989	0.982	0.988	0.982	0.988	0.98	0.989	0.973
Sample size	2424	1024	944	456	2424	1024	944	456

Annotation: 1. *p<0.1, **p<0.05, ***p<0.01;

2. Due to the limitation of space, the influence coefficient of 28 specific supports and 13 specific burnout were not included in this table;

3. NFI, RFI, IFI, TLI and CFI are indicators of model fitness. When they exceed 0.90, it indicates that the model has a good fitting effect.

Additionally, organizational support was replaced by P-O fit to compare the influence of organizational support and job fit on job burnout. The result also shows a good model fit. It shows that P-O fit also makes a significant positive impact on reducing job burnout of researchers, which plays a more important role than organizational support (-0.741<-0.661) in all career stages and three dimensions of job burnout. Meanwhile, different from organizational support, P-O fit is the most helpful for early career researchers and reduced personal accomplishment.

### Heterogeneity in eleven countries

**Fig 6:**
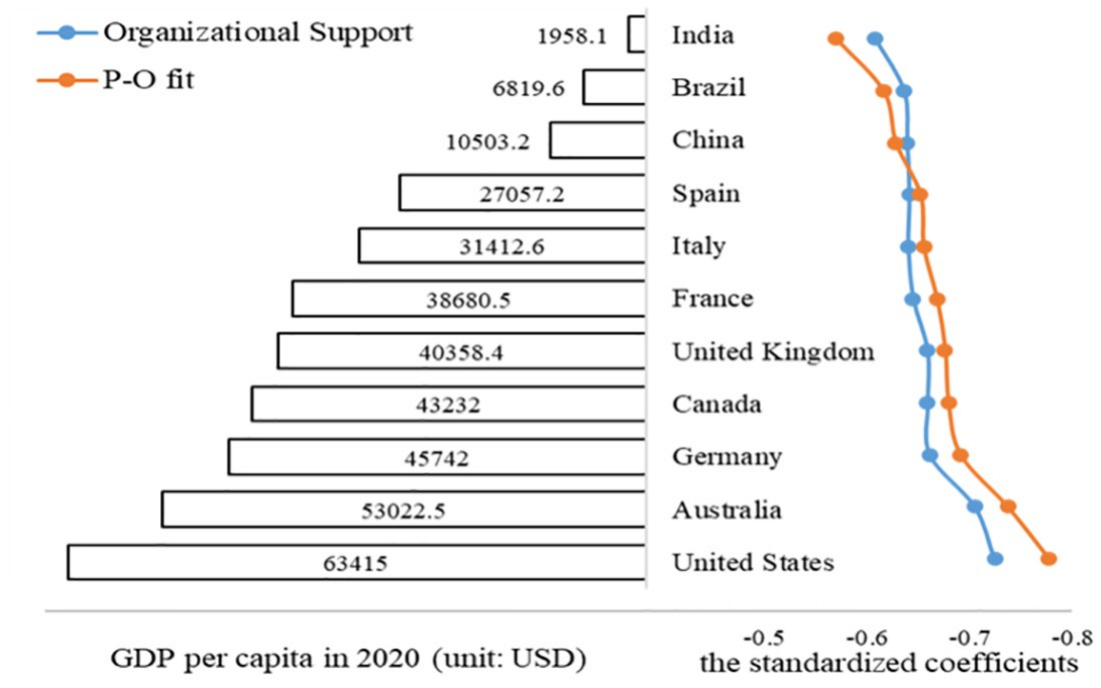
Heterogeneity of the impact of P-O fit on job burnout in eleven countries.

Further heterogeneity analysis of eleven countries showed that the influence of P-O fit is not always greater than that of organizational support in different countries. As Fig 6 shows, the standardized coefficients of the impact of organizational support and P-O fit on job burnout gradually increases with the economic development of countries. It shows that in some developing countries with low economic development (such as India, Brazil and China), the influence of organizational support is greater than that of P-O fit. However, with the development of economy, the impact of P-O fit increases rapidly. In countries with higher economic development level, P-O fit plays a greater role than organizational support.

### 28 Specific P-O fit items: Which helps more?

In 28 P-O fit items, the data show what matters most. In the path analysis, we also got the standardized regression coefficients of 28 items which can be seen as the degree of contribution of each item to job burnout, at the same time, combined with the value of each P-O fit shown in Table 4, namely the degree of fitness. In this way, 28 items can be divided into four types: Type I are less fit and helpless ones, whilst type II are less fit but helpful. Type III are those better fit and helpful while type IV are better fit but helpless ones. For researchers in different career stages and countries, Table 6 summarizes the attribution of 28 items. In order to improve the efficiency of organizational support and better assist researchers to reduce their job burnout, the organizational support in type II should be increased appropriately to meet the personal demands of researchers, while the organizational support in type IV should be reduced accordingly to avoid ineffective investment and waste of support.

**Table 6:** The degree of specific P-O fit and its contribution.

Dimensions	items	Total	Early Career	Mid-career	Late Career	United States	Australia	Germany	Canada	United Kingdom	France	Italy	Spain	China	Brazil	India
Career related	AOG	III	III	I	IV	III	IV	III	III	III	II	III	II	I	IV	IV
	ATC	IV	IV	IV	IV	IV	IV	IV	IV	IV	III	IV	IV	I	IV	II
	ATW	IV	IV	IV	I	IV	I	III	IV	IV	I	I	IV	II	IV	II
	CAO	II	II	II	II	II	II	II	II	II	II	II	II	II	II	II
	JS	I	I	I	IV	I	I	I	I	I	IV	IV	I	II	I	I
	OTW	III	III	III	III	III	IV	III	III	III	III	III	III	II	III	II
	RFA	II	II	II	II	II	II	II	II	II	II	II	II	III	III	II
	WIN	III	III	III	III	III	IV	III	III	III	III	III	III	III	IV	IV
Commitment	DRW	I	I	I	I	I	I	I	I	II	II	II	I	I	I	IV
	OCD	II	I	II	I	I	III	II	II	I	II	I	II	III	II	III
	OCE	I	I	I	II	I	I	I	I	IV	I	I	I	III	II	III
Financial	AOF	I	I	I	I	I	II	I	II	I	I	I	II	II	I	I
	BEN	IV	I	IV	IV	IV	IV	IV	III	I	IV	I	I	I	I	I
	FRO	I	IV	I	I	IV	II	IV	I	I	I	I	II	II	I	I
	SC	I	I	I	IV	I	IV	IV	I	I	I	I	I	I	I	I
Interpersonal	CWS	III	III	II	II	II	IV	II	III	III	III	II	II	III	IV	III
	RWC	III	III	III	III	IV	III	III	IV	III	IV	III	IV	IV	III	III
Physical	TSO	III	III	III	III	III	III	III	III	IV	III	III	III	III	III	III
	WFA	III	III	III	III	III	IV	IV	III	IV	IV	II	IV	III	II	III
Psychological	MOJ	III	III	III	III	III	III	III	IV	III	III	III	III	III	III	III
	PSO	II	II	III	III	II	II	II	IV	II	II	II	II	III	III	III
	WI	III	III	III	III	III	III	III	III	III	III	III	III	III	III	III
Time	AOT	I	IV	I	I	I	I	I	I	I	I	IV	I	I	I	IV
	COJ	I	I	II	IV	II	II	II	I	II	I	III	III	I	II	II
	CT	IV	IV	IV	IV	IV	IV	IV	IV	IV	IV	IV	IV	IV	IV	IV
	THW	III	III	II	III	III	II	IV	III	II	IV	IV	IV	II	III	IV
	TO	IV	IV	IV	IV	IV	IV	IV	IV	IV	IV	IV	IV	IV	III	II
	WLB	II	II	II	II	II	II	II	II	II	IV	II	III	II	II	II

Note: The specific meanings of abbreviations are shown in Table 2.

## Discussion

Underpinned by the P-O fit theory, this study examined how the P-O fit impacts job burnout of researchers in eleven countries at their different career stages during the COVID-19 pandemic. Drawn upon the data and the comprehensive analysis presented above, three main claims would be discussed in this section.

Firstly, researchers in the early and mid-career stages have higher burnout and lower P-O fit. In contrast, researchers in the late-career stage have the lowest burnout and the highest P-O fit. However, this also indirectly explains that the job burnout of researchers can be reduced by increasing P-O fit. Meanwhile, the findings would suggest that it is contrary to the job burnout process model constructed by Friedman [19] that individual job burnout is a long-term developmental process as for researchers, the job burnout does not appear in the late career stage, but is more serious in the early and mid-career stages. It is likely to be explained with two reasons: on one hand, unlike other occupations, scientific research work has its distinctive feature of high level pressure and blurred work-life boundary, which is often more demanding and challenging for researchers in their early career stage, which was supported by Lai and Li [20], who emphasized the disadvantaged situation and less sense of belongings in the collaborative research team; meanwhile, because this survey was conducted during the COVID-19 period, early and mid-career researchers are more likely to be in a process of adaptation and familiarization in the workplace, which was exacerbated by the closure of the labs and pause of the experiments caused by the pandemic to those disciplines heavily dependent upon the laboratory work [21], and when research progresses poorly, particularly when the levels of engagement in writing and publishing were not well perceived, burnout will increase. On the other hand, the late career researchers have the higher the P-O fit. Researchers in the late career stage are more likely to get accustomed to the organization and would be satisfied with the support that the organization provides. However, the P-O fit of some of the 28 items is still low in the late career stage: for example, (1) the P-O fit of researchers in CAO, RFA, DRW, OCD, FRO, AOT, WLB, etc. has always been low; (2) P-O fit in AOF keeps decreasing with the career development; (3) P-O fit in JS and OCE keeps improving cross three stages, but it is still low in the late career stage; (4) In SC, BEN, COJ, P-O fit is lower in the early career stage, but with the continuing improvement of career development, the fit degree is higher in the late career stage.

Secondly, although P-O fit has a greater inhibitory effect on job burnout than organizational support in general, the influence of P-O fit is not always greater than that of organizational support in different countries. P-O fit and its effect on job burnout presents the country or the resource-sensitive. It indicates that, when social resources are relatively scarce, it is more significant to provide more organizational support to researchers than to meet individual needs. However, when social resources are sufficient, personal needs would be focused to improve the P-O fit, which can reduce the job burnout of researchers, rather than providing organizational support with no importance.

Furthermore, this study measured the P-O fit of researchers in a relatively objective way, which would be claimed to serve as a development of Maslach’s Mismatch Model. Existing studies often implement the direct measurement by asking people explicitly whether they perceive it a good fit or not. Employers directly rated how compatible their values were with those of their organizations and how often they had to compromise personal principles to meet organizational expectations. Those who evaluated their values as highly congruent with the organization reported a variety of positive effects, such as greater feelings of personal success and higher organizational commitment than those reporting low value congruence. However, the way of direct measurement confuses people and organizational structure on one hand, and, meanwhile, hinders the estimation of its independent impact. When used in combination, it leads to a consistency bias (i.e., "I think I’m a good fit, so I must be satisfied with my job"). Therefore, the measurement framework of P-O fit in this paper can contribute to this field and complement the disadvantages of self-report measurement to a certain extent.

## Conclusion

Drawing upon the data from the Nature’s Global Career Development Survey initiated by the Nature in 2021, this paper analyzed 2,424 effective samples in the eleven countries to investigate how the P-O fit impacts researchers’ job burnout during the COVID-19 pandemic. Generally, the P-O fit of researchers sampled is 77.76%, and is highest in the late career stage (80.76%). Correspondingly, the burnout of researchers is more serious in the early and mid-career stages. However, organizational support and P-O fit all have significantly assisted researchers in all career stages to reduce the job burnout. And overall P-O fit has a greater inhibitory effect on job burnout than organizational support, though it varies with the development stages of different countries.

## Recommendation

Drawn upon these findings, the implications could be presented to shed the light on the future research policies and practices to supply and facilitate the development of researchers to make the junior talents survive during the pandemic and afterwards. Therefore, suggestions for organizations, policymakers and researchers on how to mitigate job burnout and enhance P-O fit are provided.

For organizations, when resources are relatively scarce in some developing countries, it is more important to provide more organizational support to researchers. However, when resources are sufficient in developed countries, personal needs would be focused to improve P-O fit, which would reduce the job burnout of researchers more effectively than providing organizational support blindly. Therefore, what support that organizations should provide for researchers in their career stages matters and varies in different countries. In order to improve the efficiency of organizational support and better help researchers reduce their job burnout, those less fit but helpful items should be increased appropriately, while those better fit but helpless items should be reduced appropriately to avoid ineffective investment and waste of support.

For policymakers, it is important to understand the personal needs of researchers before issuing relevant policies. Especially, it is important to pay attention to the differences in demands among different career stages. Although it is difficult to meet the needs of everyone, it is possible to understand the differences in needs in advance and coordinate when making policies. Additionally, it is necessary to understand the degree of job burnout among different researchers. For researchers with severe burnouts, more care and support should be given.

For researchers, firstly, a clear assessment of the degree of one’s own occupational burnout can be made based on the Job Burnout Scale in the research, which is helpful for the smooth progress of research work, because many researchers may not be aware of the severity of their job burnout. What’s more, it is important to present your needs in all aspects to the organization, in particular, when policies cannot cater for each individual. It is easy for organizations to overlook the needs of each individual researcher. Therefore, the sincere communication and expression of self needs becomes very important and helpful to organizations.

## Limitations and future research

Despite of the contribution of this study to the existing literature, further studies could be carried to investigate the work performance of researchers when organizational support are greater than personal demands. Although we have done a heterogeneity analysis of the actual situation in eleven countries, different countries have different policies and research contexts. Therefore, future studies can do in-depth analysis of a specific country. Given P-O fit theory, the measurement is conducted only between individual needs and organizational supply in complementary fit, namely needs-supplies fit, without discussing supplementary fit and demands-abilities fit. It is mainly due to the fact that the survey data analyzed in this study were already publicly available with their fixed items in the questionnaire. We were unable to add relevant questions to our own research design. Therefore, future research could be conducted to include the supplementary fit and demands-abilities fit of researchers. Moreover, the P-O fit calculated in this paper is mostly less than 100%, so we would not be able to make an in-depth analysis of the situation where supply is greater than demand. However, in reality, if supply is much higher than demand (when P-O fit is more than 100%), it may also have a detrimental effect on researchers. Therefore, in the future study, we will focus upon the work performance of researchers when organizational support is greater than personal demands.
